# Do experiences and perceptions about quality of care differ among social groups in Nepal? : A study of maternal healthcare experiences of women with and without disabilities, and Dalit and non-Dalit women

**DOI:** 10.1371/journal.pone.0188554

**Published:** 2017-12-19

**Authors:** Hridaya Raj Devkota, Andrew Clarke, Emily Murray, Nora Groce

**Affiliations:** 1 Department of Epidemiology and Public Health, University College London (UCL), London, United Kingdom; 2 Technical Support Unit, Kidasha UK/Nepal, London, United Kingdom; 3 Division of Epidemiology and Public Health, University College London (UCL), London, United Kingdom; Yokohama City University, JAPAN

## Abstract

**Background:**

Suboptimal quality of care and disparities in services by healthcare providers are often reported in Nepal. Experience and perceptions about quality of care may differ according to women’s socio-cultural background, individual characteristics, their exposure and expectations. This study aimed to compare perceptions of the quality of maternal healthcare services between two groups that are consistently considered vulnerable, women with disabilities from both the non-Dalit population and Dalit population and their peers without disabilities from both non-Dalit and Dalit communities.

**Methods:**

A cross-sectional survey was conducted among 343 total women that included women with disabilities, Dalits and non-Dalits. Women were recruited for interview, who were aged 15–49 years, had been pregnant within the last five years and who had used maternal care services in one of the public health facilities of Rupandehi district. A 20-item, Likert-type scale with four sub-scales or dimensions: ‘Health Facility’, ‘Healthcare Delivery’, ‘Inter-personal’ and ‘Access to Care’ was used to measure women’s perceptions of quality of care. Chi-square test and *t* test were used to compare groups and to assess differences in perceptions; and linear regression was applied to assess confounding effects of socio-demographic factors. The mean score was compared for each item and separately for each dimension.

**Results:**

All groups, women with disabilities and women without disabilities, Dalit and non-Dalit rated their perceptions and experiences of quality of care lowly in a number of items. While perceived quality of care between women with disabilities and without disabilities in the ‘Health Facility’ dimension and associated items, was found to differ (p<0.05), this difference was linked to disability status, but was not linked to caste differences. For example, differences in mean scores relating to ‘Cleanliness and Facilities’, ‘Open and Friendliness’ and ‘Compassion and Kindness’ were highly significant (p<0.001), with women with disabilities rating these as better than women without disabilities. On the other hand, women without disabilities rated the ‘Availability of cash Incentives’ more highly (p<0.01). No significant differences were found between Dalit and non-Dalit women in perceived quality of care, except in relation to ‘Cleanliness and facilities’, which Dalit women rated lower than non-Dalits (p<0.05).

**Conclusions:**

Perceptions about the quality of care differed significantly by disability status but not by caste. All groups rated the quality of healthcare delivery, interpersonal and personal factors as well as access to services ‘low.’ Poor service user experiences and perceptions of quality of care undermine opportunities to translate increased healthcare coverage into improved access and outcomes. Greater attention is required by policy makers, health planners and providers to the improvement of quality of care in health facilities.

## Introduction

Provision of quality healthcare and utilization of health services during pregnancy, childbirth and in the post-natal period are important contributors to woman’s maternal health outcomes. There is growing consensus that much maternal and new-born mortality could be prevented with the provision of better quality care [1–4]. Numerous studies of maternal healthcare services in Asia have found that both public and private health facilities offer sub-optimal services, with short-comings in the physical facilities and the quality of maternal healthcare provided [5,6]. Disparities in services by healthcare providers are often reported [7], and the experience and perception of care may differ according to women’s background characteristics, previous exposure and expectations. There is also a growing body of evidence to show that women’s perception of quality of care is one of the key determinants of service utilization although this continues to be an often neglected aspect of assessing access to care in low and middle-income countries [8,9]. User’s perception could also be affected by their personal, social and cultural circumstances [10]. Similar findings are reported specifically from Nepal [11–13]. However, the experience of user’s perceptions when they are members of historically vulnerable subgroups and how these differ from the general population of women, has been little explored.

The literature reports that both women with disabilities—defined here as those who have long-term physical, mental, intellectual or sensory impairments which in interaction with various barriers may hinder their full and effective participation in society on an equal basis with others [14], and Dalit women face numerous barriers in accessing maternal healthcare services due to their lower status in the society. ‘Dalits’ are considered ‘untouchables’ and are at the bottom of the caste hierarchy and the word literary means oppressed. In Nepal, women with disabilities and women from Dalit caste groups have low rates of maternal health service utilization [15,16]. This study attempts to better understand the perceived quality of care and experience of maternal healthcare services of these two groups both commonly regarded in Nepal as being socially discriminated and oppressed.

## Background

Over the past two decades, Nepal has made good progress towards improving maternal health outcomes. There has been a significant increase in utilization of maternal healthcare services and the maternal mortality ratio has fallen dramatically to 258/100,000 in 2015 from 548/100,000 in 2000 [17]. However increases in utilisation have not been equitable, with persistent disparities in utilization among different population groups [11,15,18,19].

High coverage alone is not enough to reduce mortality and needs to be accompanied by improved quality throughout the continuum of care [3,4,20]. Good quality maternal healthcare in pregnancy and childbirth is a basic reproductive right that should be ensured by the state [21,22]. In recent decades, attempts have been made to drive more equitable improvement of maternal outcomes in low and middle-income countries, but substantial inequalities in care quality remain in many countries. Disproportionate resource allocation, poorly developed health systems and socio-cultural, economic and physical factors are all recognised as factors leading to health disparities and poor maternal outcomes [23,24].

Poor quality of care is a contributing factor in both public and private health facilities in low-income countries [12,25,26]. The capacity and number of health workers, quality and availability of drugs, low expectations of care, short opening hours and lack of privacy during antenatal care and delivery are frequently cited as maternity care concerns in Nepal; however, women with disabilities appear particularly vulnerable to experiencing distress caused by these factors [8,16,25].

Staff attitudes, demonstrated by being friendly, polite, welcoming, respectful, sympathetic, non-discriminatory and protecting the confidentiality of mothers are key factors affecting clients’ perceptions of care quality. Similarly, the competence and efficiency of health workers are reported to influence user’s perception of care; as do factors about the health facility environment such as accessibility, space, hygiene and sanitation, comfort and availability of supplies [27–29].

The experience of care can vary by the background characteristics of a woman and her family. Socio-demographic factors such as level of education, distance to a health facility and mode of transport to reach services are linked to a client’s perception of care [30,31]. Illiterate women with low social status and restricted personal freedoms are reported to have lower expectations of care quality than do their educated, higher status counterparts [32]. It is also reported that health providers sometimes fail to treat women from ‘lower’ social groups with respect during their labour and childbirth [33]. Further, disparities are reported between the information given to Muslim and Hindu women by health providers during their ante natal check-ups [26]. Poor quality of care in both government and private facilities was commonly reported by women with disabilities in health services [34], whilst others report no differences in services given to women with and without disabilities [35].

Quality of care is a complex and multidimensional concept [20] and there is no universally accepted definition. Some define ‘‘quality as excellence to expectations or goals, which have been met” [36]; others define it as ‘‘fitness-for-purpose” [37] or ‘‘the degree to which health services for individuals and population increase the likelihood of desired health outcome and are consistent with current professional knowledge” [38,39]. WHO defines quality of care as ‘‘the extent to which healthcare services provided to individuals and patient populations improve desired health outcomes. In order to achieve this, healthcare needs to be safe, effective, timely, efficient, equitable and people-centred” [40]. Based on the WHO definition, quality of care in maternal healthcare considers two important components: the quality of provision of care and the experience of women, new-borns and their families.

The WHO recommended quality of care framework [4] contains eight dimensions of care and recognises quality of care as being a core component of the right to health. However, this study focused on user’s experience and is limited to four dimensions. Few studies about women’s perspective of health services have been undertaken in Nepal and even less is known about the experience of maternal healthcare services of vulnerable populations such as women with disabilities and Dalit women.

### Objective

To compare perceptions of the quality of maternal healthcare services between women with disabilities and women without disabilities, and Dalit and non-Dalit women in a southern district of Nepal.

## Methods

### Setting

The study was conducted in Rupandehi, a southern district of Nepal with a population of 880,196 of which 50.89% are female [41]. The majority of people (78%) live in rural villages, but the urban population is growing fast. 86% are Hindu, 8% Muslim and 5% Buddhist; and the population comprises upwards of 125 different ethnic and indigenous groups, including 12.03% Dalits, 24.45% Janajatis, 15.21% Brahmins and 5.62% Chhetries. Dalits are considered untouchables and the lowest group in the social hierarchy. Only 1.12% population in the study district are reported to have a disability as per the government census [41].

Medical officers, nurses, auxiliary nurse midwives, and the other paramedics such as health assistants and auxiliary health workers deliver primary healthcare services including maternal healthcare through five Primary Healthcare Centres (PHCC), six Urban Health Clinics (UHC), six Health Posts (HP) and 58 Sub-Health Posts (SHP) distributed across the district. SHPs, HPs and UHCs are staffed with three to four paramedics whereas PHCCs are staffed with ten higher-level technical personnel including nurses and a medical officer. One district hospital and one zonal hospital (covering six districts) provide secondary care services in the district. In addition to the government health sector, there is also a wide network of NGOs and private sector services with private hospitals, nursing homes, clinics and pharmacies/drug shops [42].

### Study design

A cross-sectional survey was conducted in 2014 among married and unmarried women aged between 15–49 years, who had given birth within the last five years. Recruitment was limited to women who had utilized any government health facilities for antenatal care or delivery during their last pregnancy, primarily because maternal care is free in government health services, making it more affordable for people with disabilities and Dalit people and those from poorer socio-economic backgrounds. Government delivers most routine and acute health care in Nepal and approximately 70% of the population use these services each year, the private sector providing a smaller proportion of mainly urban acute services [18].

### Sample

The study used a multi-stage sampling method for selection and recruitment of participants. As no specific list of people with disabilities exists in the district, in the first stage, women with disabilities within the criteria were identified using the most recent census and district reports produced by government, NGOs and local disabled people’s organizations (DPOs), and these were listed in the sampling frame. Out of 119 women with a disability identified and listed, 89 were located and 68 women selected for interview using study inclusion criteria and the disability screening criteria of the UN Washington Group on Disability Criteria (Short set)–S1 Annex (www.washingtongroup-disability.com/) [43]. Considerable efforts, enquiries and time on field visits was spent trying to locate the other 30 women originally identified, but without success. The challenges in locating women with disability and securing a sample that met the Washington Group criteria, presented potential difficulties to achieving an overall sample size large enough for statistical testing. Therefore, the number of women with disabilities, Dalit women and non-Dalit women without disabilities needed for interviews was determined using a 1:2:2 ratio respectively, with stratified random sampling used for selection of Dalit and non-Dalit women. Female Community Health Volunteers (FCHVs), community leaders and representatives of local NGOs and DPOs helped in identification, consultation and recruitment of participants. In total 343 women distributed across the district were interviewed and included for analysis having used any one or multiple government health facilities. There was no attempt to identify individual health facilities, as the focus was on understanding women’s common experience. Furthermore, the complexity of separating and attributing different experiences to one of many different settings, within an environment with no routine sequence of utilising health facilities of different levels (women can go to any level of health facility at any time) was beyond the scope of this study. However, the distribution and number of participants suggest that the majority of health facilities in the district were utilised by participants.

### The tool and data collection

A 20-item Likert-type ‘five points’ rating scale tool was used for data collection (S2 Annex). The tool was initially developed for use in Vietnam [6] and was used in Nepal with adjustments for assessing the perceived quality of maternal care services [12]. The tool was adjusted to match the local context, the final version consisting of 20-items in four dimensions. The Cronbach’s alpha confirmed internal consistency of the scale, which was 0.78 for the whole scale ranging 0.75 to 0.79 for each item.

Dimension A consisted of seven items about ‘Health Facilities’; dimension B consisted of five items about ‘Healthcare Delivery’; dimensions C and D consisted of four items each about ‘Inter-personal Aspects of Healthcare Providers’ and ‘the Accessibility of Services’. The tool, with priori chosen dimensions, was translated into the Nepali language and field-tested before wider administration.

Twelve field data collectors were trained and the authors monitored the interview and data-collection process. Completed forms were checked; any found incomplete or with entry errors were identified and participants revisited to complete or confirm the information. The interviewers read aloud each statement in the tool in Nepali and recorded the participant’s response according to a five-point Likert scale. The interview and questions were conveyed in local sign language for participants with hearing disabilities and a mood chart with five emotional (happy to weeping) faces was used as a support tool to facilitate rating the level of agreement or disagreement.

Ethical approvals for the study were obtained from University College London (UCL)–Project ID: 5260/001 and from the Nepal Health Research Council (NHRC)–Ref. No. 1184. Verbal and signed consent was taken from all participants before conducting interviews. Guardian’s consent was also obtained for participants with intellectual disability.

### Variables and description of measures

Women’s perception and experience of quality of care were the outcome variables investigated. The variables of interest in the study were women’s disability and caste, which were taken as primary predictors. Other independent variables selected were age of women, place of residence, religion, level of education, marital status and number of pregnancies (parity). “S1 Table” shows study variables, variable coding and description of variables.

The ratings of the tool were coded as: strongly agree = 2, agree = 1, neither agree nor disagree = 0, disagree = -1 and strongly disagree = -2. The tool consisted of nine negative items (statements) (A: 3, 7; B: 2, 4, 5; C: 3, 4; D: 1, 3). The negatively coded statements were reverse coded before analysis. Mean scores were compared for each item and for different dimensions to assess differences in perceptions among study groups.

### Statistical analysis

Interview data from 343 participants were included for analysis. Data was cleaned and checked, before being entered using Epi-Data 3.1 to minimize entry error, and imported into SPSS version 22.0 for analysis. We conducted bivariate and multivariate statistical analysis. Chi-square tests were used to explore bivariate associations between characteristics, disability, and caste status. Outcome variables were measured by computing the average perception scores for women based on the 20 items. Initially, sample characteristics of study participants were compared by disability and caste status, separately, through chi-square tests. Next, independent sample t tests were used to examine the perception differences between women with disabilities and without disabilities and Dalit and non-Dalit women. Linear regression was conducted to control for confounding effects in the differences. The overall average women’s perceived quality proxy indicator was created by summing scores of all 20-items as a dependent variable. Control variables included in the regression models were respondent’s disability, caste, age, place of residence, religion, level of education, marital status and parity. All control variables were checked for collinearity among variables using a collinearity diagnostic test and none had a variance inflation factor (VIF) more than 1.508.

## Results

### Demographic and descriptive statistics

“S2 Table” shows over half the women (51%) were Dalit and about one-fifth (20%) had a disability. Among the participants with disabilities, a quarter also were Dalit, two-third (68%) had a physical disability, one-fifth (22%) a visual disability, and a few had hearing (6%) or multiple (4%) disabilities. About half of the 343 participants were aged between 25–34 years, two-fifths were below 25, and one in ten were over 34 years old. Two-thirds of participants were also multipara. The vast majority of the participants (78%) lived in rural areas, including all of the Dalit women with disabilities. Two-fifths of women reported themselves as illiterate, while one-third stated education up to primary level and approximately a quarter to secondary level or above. More than 80% of participants were Hindu. In bivariate analysis, the association between participant’s disability status and age distribution, place of residence and education was statistically significant (P<0.05). In the case of caste status, this association was significant only with the place of residence (P<0.05).

### Perceptions of women with disabilities and without disabilities about quality of care

Out of the four dimensions, mean scores of perceived quality were only higher in women with disabilities for factors relating to the ‘‘Health Facility” (mean differences -1.062 and 95% CI -2.00, -0.12). However, statistically significant differences in scores were seen between women with disabilities and women without disabilities in ‘‘Cleanliness and Facilities” (mean difference -0.586 and 95% CI -0.86, -0.32), ‘‘Open and Friendliness” (mean difference -0.346 and 95% CI -0.50, -0.19), ‘‘Compassionate and Kindness” (mean difference -0.346 and 95% CI -0.50, -0.19) and ‘‘Availability of cash Incentives” (mean difference 0.476 and 95% CI 0.19, 0.76) (S3 Table). We therefore show perceived quality scores for women with disabilities compared to women without disabilities through linear regression. Socio-demographic (age, education, place of residence etc.) explained the relationship.

Perceived quality of care for Dalits, compared to non-Dalits, showed a significant difference in the mean score of ‘‘Cleanliness and Facilities” in individual items only (mean difference -0.254 and 95% CI -0.47, -0.04). There were no significant mean differences in the scores found in other items at the individual or subscale (dimension) level between Dalits and non-Dalits (S4 Table).

“S5 Table” showed no effect of women’s disability and caste status alone in perceived quality scores (P>0.05). However, disability status together with caste and education was statistically significant (P<0.05). None of the other socio-demographic variables neither alone nor together with other variables found influencing in perception score (P>0.05).

## Discussion

Women with disabilities were more positive about factors relating to ‘the Health Facilities’ (such as availability of staff and equipment, opening times, facilities and cleanliness), than women without disabilities (mean score of 7.68 compared to 6.61); but this was the only difference in perceived quality across the four dimensions of ‘Health Facility’, ‘Healthcare Delivery’, ‘Interpersonal Aspects’, and ‘Access to Services’. There was a significant difference in the perception of cleanliness, with women with disabilities rating this more highly. The reasons for this more favorable perception are unclear, but the evidence suggests that women with disabilities may be at increased risk of being socially excluded and vulnerable, which may affect their confidence to criticize services. Those with disabilities may be more reliant upon the help of the health facility and thus may also be less likely to be overtly critical. Recent research into ‘‘why health service users in Nepal do not complain” also found that despite being aware of major weaknesses, very few people complained or were openly critical, and that poorer and less empowered groups were least likely to complain [44]. Women with disabilities are frequently poorer than women without disabilities and as a result may live in worse housing conditions with which to compare the health facility. Studies in India, Nigeria, Tanzania, Ghana and Zambia have also found that women’s increasing education levels negatively affect their satisfaction with maternal care; and women with disabilities are frequently less educated than the mainstream population, so this may also contribute to their expectations and more positive satisfaction with services [45].

There were no differences in perceived quality of care between Dalit and non-Dalit women, except in relation to cleanliness and facilities’. Dalit women perceived the quality of this as being lower than non-Dalit women (0.79 vs 1.04). It is possible that the health facilities attended by more Dalit women were lower level facilities in poorer communities, that may have had limited utilities and sanitation. There were no differences in the perceived quality of ‘Healthcare Delivery’, which included items such as staff skills and training, drugs and supplies, dignity and privacy. Perceptions of ‘Inter-personal Aspects’ of care were different between women with and without disabilities in relation to individual items but not once collated at the overall ‘dimension level’; whilst the difference in perceptions of being ‘Open and Friendly’ and ‘Compassionate and Kind’ were highly significant and health worker’s attitude and behaviour seemed to have an impact on user’s perception of quality of care.

Interestingly, service users may interpret health worker’s attitudes and behaviours towards them as proxies for quality, rather than their actual knowledge and competence, an acknowledgment perhaps that clinical knowledge and competence is unlikely to translate into optimal quality of care (or outcome) in the absence of positive attitudes and supportive behaviours. This is also recognised in new global frameworks for improving quality of maternal and newborn care, that increasingly focus equally on the ‘provision of care’ and the ‘experience of care’ [4]. Women with disabilities rated health worker attitudes and behaviours more positively than women without disabilities, which is contrary to findings of previous studies reporting disparities in experiences and negative attitudes towards women with disabilities, as well as evidence of abuse and disrespect by healthcare workers. Some studies have also found that women with disabilities are not alone in reporting such violations and this phenomena was not necessarily related to disability [16].

It may also be that the positive perceptions and healthcare experiences of women with disabilities in the study could be the result of benevolent paternalism of local health providers rather than a reflection of equality, respect or positive regard. The fact that all participants with disabilities lived in rural areas may have influenced their positive perception, as more may have received care from local facilities where health workers already know them, rather than in larger urban centers where pre-existing relationships between health worker and patient are less likely. It is also possible that the relative positivity of service users with disabilities could be due to lower expectations, the result of limited exposure to alternatives and poor previous experiences.

There was no tangible difference in the Access dimension and this was the lowest rated by all groups, concurring with a previous study in Nepal [16]. However, there was a significant difference relating to ‘travel reimbursement and delivery incentives’. Women without disabilities perceived and rated this item more highly than women with disabilities. This may be because even with travel incentives, the transportation costs for women with disabilities were greater than the funds available. For example, women with physical disabilities unable to take the bus, may have to pay for more costly taxi services.

Although it was anticipated that there would be significant differences in perceptions of quality of care between vulnerable and non-vulnerable groups, there were in fact only a limited number of differences reported between women with and without disabilities, and even fewer between Dalit and non-Dalit women. The study found fewer associations between socio-demographic factors and perceived quality of care than studies in other settings [46,47]. However, it is important to note that all groups rated many aspects of care very low and thus the findings in this study reflect overall dissatisfaction with the quality of care. Also few of the women in this study had other experiences with which to compare the care they received locally upon which to base an informed view about the adequacy of equipment, staff skills or training; or to set their levels of expectation of services.

Poor experiences of care can deter use of health services [29], so if Nepal is to build on the progress made in recent years to increase utilization and reduce mortality, then quality and experience of care need to be better understood and improved upon, including indirect factors that may impact this such as transportation, distance to facilities or the preparation, pay and working conditions for health workers. Health provider’s (nurse’s) job satisfaction may also be related to service user’s perception of quality of care [48]. In the Nepali context, low pay and poor working conditions including inadequate physical infrastructure, erratic supplies of equipment and drugs, inequitable management approaches and poor housing, have been found to affect the job satisfaction of health providers and thus may contribute to adverse experiences and perceptions of quality of care [49].

### Limitations

We acknowledge limitations associated with this study. The study findings may not be generalizable since the study sample was limited to one southern district. While a range of women with different types of disabilities and all Dalit ‘sub-caste’ groups were included in the study, there was exclusion of a small number of women with very severe communication difficulties (due to either severe intellectual disability or a hearing disability with no signing or other routes of communication) and this may affect generalizability of the findings to all women with disabilities. This is of particular relevance because some literature shows that individuals with hearing disabilities or those who have intellectual impairments may be at increased risk for being excluded and being victims of violence and abuse, even in comparison to individuals with other types of disabilities [50]. Additionally, we recruited women who had their last pregnancy within five years, so we also recognize the potential for recall bias. Finally, we found that a number of women interviewed sought care from more than one health facility during their pregnancies, so in some cases, it was unclear which of the facilities providing care the women surveyed were reporting on.

## Conclusion

Perceived quality of care differed significantly in several dimensions between women with and without disabilities, however little difference was found between women of different castes. Across the four dimensions and twenty items, women rated their experience of care as being low in almost all aspects, irrespective of caste or disability. This could have an impact on the progress Nepal has made in increasing utilization of health services and reducing maternal mortality, and prevent consolidation of these trends. Further research is necessary to explore differences and consistencies in perceptions and experiences of care across other regions and between different social groups and service users, and future research which combines the satisfaction tool with a Likert tool would provide further insight into quality of care.

Positive experiences of care are associated with greater willingness to use health facilities again in future [29]. It is vital therefore, that efforts are made to better understand the causes of poor experiences and quality of care, and comprehensive steps taken to address these.

**Declaration:** The views expressed in this article are those of the authors and do not necessarily represent the views of agencies supporting this study.

## Supporting information

S1 Table Coding and description of variables. (DOCX)

S2 Table Characteristics of respondents by disability status and caste. (DOCX)

S3 Table Mean differences (95% CI) in perceived quality of care item scores by disability status. (DOCX)

S4 Table Mean differences (95% CI) in perceived quality of care item scores by caste. (DOCX)

S5 Table Adjusted mean differences in perceived quality scores by disability status (n = 343). (DOCX)

S1 ANNEX UN Washington Group Disability Criteria (Short set). (DOCX)

S2 ANNEX Perception survey tool (20-items five rating scale Likert type). (DOCX)
