# Supplementary material for: Do experiences and perceptions about quality of care differ among social groups in Nepal? : A study of maternal healthcare experiences of women with and without disabilities, and Dalit and non-Dalit women
Source: PLoS One. 2017 Dec 19;12(12):e0188554. doi: 10.1371/journal.pone.0188554 (PMC5736179; doi:10.1371/journal.pone.0188554)
Supplement: S1 Table — (DOCX) [file pone.0188554.s001.docx]

**Table 1: Coding and description of variables**

| **Variable** | **Coding** | **Description** |
| --- | --- | --- |
| **Socio-Demographic** |  |  |
| Age of women | Continuous | Completed age of the woman at the time of survey in years |
| Disability status | 1 = Women without disabilities | Women’s meeting UN Washington Group disability criteria |
|  | 2 = Women with disabilities |  |
| Caste | 1 = Dalit | Woman's reported caste after marriage |
|  | 2 = Non-Dalit |  |
| Place of residence | 1 = Rural | Woman's place of residence at the time of survey |
|  | 2 = Urban |  |
| Religion | 1 = Hindu | Self-reported religion of the woman |
|  | 2 = Others |  |
| Respondent's education | 0 = Illiterate | Number of years of education completed by the woman |
|  | 1 = Primary (up to 5 grade) |  |
|  | 2 = Secondary and above (6 +) |  |
| Marital status | 1 = Married | Woman living together with partner at the time of survey |
|  | 2 = Unmarried/Single |  |
| Parity | 1 = Primipara (Single Pregnancy) | Number of live births till the date of survey |
|  | 2 = Multipara (Multiple Pregnancies) |  |
