# Supplementary material for: Do experiences and perceptions about quality of care differ among social groups in Nepal? : A study of maternal healthcare experiences of women with and without disabilities, and Dalit and non-Dalit women
Source: PLoS One. 2017 Dec 19;12(12):e0188554. doi: 10.1371/journal.pone.0188554 (PMC5736179; doi:10.1371/journal.pone.0188554)
Supplement: S2 Table — (DOCX) [file pone.0188554.s002.docx]

**Table 2: Characteristics of respondents by disability status and caste**

|  | ***Women with disabilities** | **Women without disabilities** |  | **Dalit** | **Non-Dalit** |  |
| --- | --- | --- | --- | --- | --- | --- |
| **Characteristics** | **n=68 (19.8%)** | **n=275**  **(80.2%)** | **P - value** | **n=174 (50.7%)** | **n=169 (49.3%)** | **P - value** |
| **Respondent's Age** |  |  |  |  |  |  |
| <25 | 12 (17.6%) | 118 (42.9%) | P=0.000 | 65 (37.4%) | 65 (38.5%) | P=0.857 |
| 25 - 34 | 36 (52.9%) | 135 (49.1%) |  | 89 (51.1%) | 82 (48.5%) |  |
| >34 | 20 (29.4%) | 22 (8%) |  | 20 (11.5%) | 22 (13%) |  |
| **Place of Residence** |  |  |  |  |  |  |
| Rural | 64 (94.1%) | 205 (74.5%) | P=0.000 | 127 (73%) | 142 (84%) | P=0.013 |
| Urban | 4 (5.9%) | 70 (25.5%) |  | 47 (27%) | 27 (16%) |  |
| **Religion** |  |  |  |  |  |  |
| Hindu | 58 (85.3%) | 234 (85.1%) | P=0.966 | 146 (83.9%) | 146 (86.4%) | P=0.518 |
| Non-Hindu (Other) | 10 (14.7%) | 41 (14.9%) |  | 28 (16.1%) | 23 (13.6%) |  |
| **Education** |  |  |  |  |  |  |
| Illiterate | 27 (39.7%) | 110 (40%) | P=0.022 | 69 (39.71%) | 68 (40.2%) | P=0.711 |
| Primary (up to 5 grade) | 14 (20.6%) | 96 (34.9%) |  | 59 (33.9%) | 51 (30.2%) |  |
| Secondary & higher (6 & above) | 27 (39.7%) | 69 (25.1%) |  | 46 (26.4%) | 50 (29.6%) |  |
| **Marital Status** |  |  |  |  |  |  |
| Married | 63 (92.6%) | 275 (100%) | P=0.000 | 174 (100%) | 164 (97%) | P=0.022 |
| Unmarried | 5 (7.4%) | 0 |  | 0 | 5 (3%) |  |
| **Parity** |  |  |  |  |  |  |
| Primi | 19 (27.9%) | 92 (33.5%) | P=0.384 | 55 (31.6%) | 56 (33.1%) | P=0.763 |
| Multi | 49 (72.1%) | 183 (66.5%) |  | 119 (68.4%) | 113 (66.9%) |  |

Chi square test of significance *Physical disability (n=46), Low-vision/Blind (n=15), Hearing disability (n=4), Multiple (n=3)
