# Supplementary material for: Do experiences and perceptions about quality of care differ among social groups in Nepal? : A study of maternal healthcare experiences of women with and without disabilities, and Dalit and non-Dalit women
Source: PLoS One. 2017 Dec 19;12(12):e0188554. doi: 10.1371/journal.pone.0188554 (PMC5736179; doi:10.1371/journal.pone.0188554)
Supplement: S3 Table — (DOCX) [file pone.0188554.s003.docx]

**Table 3: Mean differences (95% CI) in perceived quality of care item scores by disability status**

|  | **Disability status** | | | | |  |
| --- | --- | --- | --- | --- | --- | --- |
|  | **Women with disabilities (n=68)** | |  | **Women without disabilities (n=275)** | | **95% CI for Mean**  **Difference** |
| **Dimensions/Items** | **Mean** | **SD** |  | **Mean** | **SD** |  |
| **A. Health Facility*** | **7.68** | **3.67** |  | **6.61** | **2.61** | **-1.062 (-2.00, -0.12)** |
| Staff adequacy | 1.10 | 0.83 |  | 1.04 | 0.46 | -0.067 (-0.28, 0.14) |
| Staff availability | 1.16 | 0.77 |  | 1.01 | 0.52 | -0.154 (-0.35, 0.04) |
| System and honesty | 0.81 | 1.21 |  | 0.81 | 1.20 | -0.002 (-0.32, 0.32) |
| Enough rooms and space | 1.10 | 0.95 |  | 0.99 | 0.69 | -0.110 (-0.35, 0.13) |
| Equipment and materials | 1.16 | 0.84 |  | 1.01 | 0.62 | -0.147 (-0.36, 0.07) |
| Cleanliness and facilities*** | 1.38 | 0.77 |  | 0.80 | 1.06 | -0.586 (-0.86, -0.32) |
| Health facility opening time | 0.96 | 1.03 |  | 0.96 | 1.07 | 0.004 (-0.28, 0.29) |
| **B. Healthcare Delivery** | **4.96** | **3.24** |  | **4.71** | **2.93** | **-0.243 (-1.04, 0.55)** |
| Service standards | 1.25 | 0.90 |  | 1.10 | 0.60 | -0.148 (-0.38, 0.08) |
| Staff skill and training | 0.75 | 1.01 |  | 0.72 | 0.99 | -0.026 (-0.29, 0.24) |
| Drugs and supplies | 1.19 | 0.87 |  | 1.09 | 0.52 | -0.097 (-0.32, 0.12) |
| Dignity and privacy | 1.03 | 1.13 |  | 0.85 | 1.26 | -0.179 (-0.49, 0.13) |
| Service procedure | 0.74 | 1.09 |  | 0.94 | 1.03 | 0.207 (-0.07, 0.48) |
| **C. Inter-personal Aspect** | **4.10** | **2.79** |  | **3.49** | **2.44** | **-0.608 (-1.28, 0.06)** |
| Open and friendly*** | 1.40 | 0.58 |  | 1.05 | 0.60 | -0.346 (-0.50, -0.19) |
| Compassionate and kindness*** | 1.40 | 0.58 |  | 1.05 | 0.60 | -0.346 (-0.50, -0.19) |
| Welcoming and respectful | 0.78 | 1.06 |  | 0.75 | 1.03 | -0.034 (-0.31, 0.24) |
| Time given by the provider | 0.53 | 1.22 |  | 0.65 | 1.08 | 0.118 (-0.20, 0.44) |
| **D. Access to services** | **1.96** | **2.40** |  | **1.90** | **2.74** | **-0.047 (-0.76, 0.67)** |
| Availability of cash incentive** | 0.34 | 0.97 |  | 0.81 | 1.09 | 0.476 (0.19, 0.76) |
| Transport access to health facility | 0.65 | 1.08 |  | 0.43 | 1.09 | -0.218 (-0.51, 0.07) |
| Distance to health facility | 0.24 | 1.29 |  | 0 | 1.28 | -0.235 (-0.58, 0.11) |
| Accommodative health facility infrastructures | 0.74 | 1.06 |  | 0.67 | 0.79 | -0.070 (-0.30, 0.16) |

*p<0.05. **p<0.01. ***p<0.001
