# Supplementary material for: Do experiences and perceptions about quality of care differ among social groups in Nepal? : A study of maternal healthcare experiences of women with and without disabilities, and Dalit and non-Dalit women
Source: PLoS One. 2017 Dec 19;12(12):e0188554. doi: 10.1371/journal.pone.0188554 (PMC5736179; doi:10.1371/journal.pone.0188554)
Supplement: S4 Table — (DOCX) [file pone.0188554.s004.docx]

**Table 4: Mean differences (95% CI) in perceived quality of care item scores by caste**

|  | **Caste** | | | | |  |
| --- | --- | --- | --- | --- | --- | --- |
|  | **Dalit (n=174)** | |  | **Non-Dalits (n=169)** | | **95% CI for Mean Difference** |
| **Dimensions/Items** | **Mean** | **SD** |  | **Mean** | **SD** |  |
| **A. Health Facility** | **6.90** | **2.80** |  | **6.75** | **2.96** | **0.145 (-0.47, 0.76)** |
| Staff adequacy | 1.08 | 0.49 |  | 1.02 | 0.61 | 0.063 (-0.06, 0.18) |
| Staff availability | 1.06 | 0.56 |  | 1.02 | 0.60 | 0.040 (-0.08, 0.16) |
| System and honesty | 0.92 | 1.13 |  | 0.69 | 1.27 | 0.227 (-0.03, 0.48) |
| Enough rooms and space | 1.01 | 0.82 |  | 1.02 | 0.68 | -0.018 (-0.18, 0.14) |
| Equipment and materials | 1.04 | 0.69 |  | 1.05 | 0.65 | -0.007 (-0.15, 0.14) |
| Cleanliness and facilities* | 0.79 | 1.14 |  | 1.04 | 0.90 | -0.254 (-0.47, -0.04) |
| Health facility opening time | 1.01 | 1.06 |  | 0.91 | 1.07 | 0.095 (-0.13, 0.32) |
| **B. Healthcare Delivery** | **4.77** | **2.85** |  | **4.75** | **3.13** | **0.019 (-0.62, 0.65)** |
| Service standards | 1.12 | 0.66 |  | 1.14 | 0.69 | -0.021 (-0.17, 0.12) |
| Staff skill and training | 0.71 | 0.96 |  | 0.75 | 1.03 | -0.033 (-0.24, 0.18) |
| Drugs and supplies | 1.09 | 0.58 |  | 1.14 | 0.63 | -0.044 (-0.17, 0.08) |
| Dignity and privacy | 0.89 | 1.25 |  | 0.88 | 1.23 | 0.009 (-0.25, 0.27) |
| Service procedure | 0.95 | 1.03 |  | 0.85 | 1.05 | 0.108 (-0.11, 0.33) |
| **C. Inter-personal Aspect** | **3.64** | **2.42** |  | **3.59** | **2.64** | **0.058 (-0.48, 0.60)** |
| Open and friendly | 1.06 | 0.66 |  | 1.18 | 0.54 | -0.114 (-0.24, 0.01) |
| Compassionate | 1.06 | 0.66 |  | 1.18 | 0.54 | -0.114 (-0.24, 0.01) |
| Welcoming and respectful | 0.79 | 1.01 |  | 0.71 | 1.07 | 0.083 (-0.14, 0.30) |
| Time given by the provider | 0.72 | 1.02 |  | 0.52 | 1.19 | 0.203 (-0.03, 0.44) |
| **D. Access to services** | **2.07** | **2.64** |  | **1.76** | **2.71** | **0.317 (-0.25, 0.89)** |
| Availability of cash incentive | 0.74 | 1.08 |  | 0.7 | 1.09 | 0.043 (-0.19, 0.27) |
| Transport access to health facility | 0.49 | 1.06 |  | 0.45 | 1.12 | 0.045 (-0.19, 0.28) |
| Distance to health facility | 0.10 | 1.29 |  | -0.01 | 1.29 | 0.115 (-0.16, 0.39) |
| Accommodative health facility and infrastructures | 0.74 | 0.80 |  | 0.62 | 0.89 | 0.114 (-0.07, 0.29) |

*p<0.05.
