# Supplementary material for: Do experiences and perceptions about quality of care differ among social groups in Nepal? : A study of maternal healthcare experiences of women with and without disabilities, and Dalit and non-Dalit women
Source: PLoS One. 2017 Dec 19;12(12):e0188554. doi: 10.1371/journal.pone.0188554 (PMC5736179; doi:10.1371/journal.pone.0188554)
Supplement: S5 Table — (DOCX) [file pone.0188554.s005.docx]

**Table 5: Adjusted mean differences in perceived quality scores by disability status (n=343)**

|  | **Model 1: Disability status only** | | **Model 2: + Caste** | | **Model 3: + Demographics** | | **Model 4: + Socioeconomic** | | **Model 5: Final model** | |
| --- | --- | --- | --- | --- | --- | --- | --- | --- | --- | --- |
| **Factors** | **Mean difference** | **95% CI** | **Mean difference** | **95% CI** | **Mean difference** | **95% CI** | **Mean difference** | **95% CI** | **Mean difference** | **95% CI** |
| **Disability status** |  |  |  |  |  |  |  |  |  |  |
| Women with disabilities | 1.960 | -0.21, 4.13 | *2.255 | 0.02, 4.49 | 1.588 | -0.75, 3.93 | *2.220 | 0.03, 4.41 | 2.203 | -0.26, 4.66 |
| Women without disabilities (Ref) |  |  |  |  |  |  |  |  |  |  |
| **Caste** |  |  |  |  |  |  |  |  |  |  |
| Dalit |  |  | 0.973 | -0.81, 2.75 |  |  |  |  | 1.014 | -0.77, 2.80 |
| Non-Dalit (Ref) |  |  |  |  |  |  |  |  |  |  |
| **Respondent's Age** |  |  |  |  |  |  |  |  |  |  |
| <25 yrs (Ref) |  |  |  |  |  |  |  |  |  |  |
| 25 - 34 yrs |  |  |  |  | 0.43 | -1.66, 2.51 |  |  | 0.571 | -1.53, 2.67 |
| >34 yrs |  |  |  |  | -0.22 | -3.44, 3.00 |  |  | 0.107 | -3.18, 3.39 |
| **Place of Residence** |  |  |  |  |  |  |  |  |  |  |
| Rural |  |  |  |  | 1.485 | -0.65, 3.62 |  |  | 1.512 | -0.70, 3.73 |
| Urban (Ref) |  |  |  |  |  |  |  |  |  |  |
| **Religion** |  |  |  |  |  |  |  |  |  |  |
| Hindu |  |  |  |  | -2.306 | -4.75, 0.14 |  |  | -2.172 | -4.66, 0.31 |
| Non-Hindu (Ref) |  |  |  |  |  |  |  |  |  |  |
| **Education** |  |  |  |  |  |  |  |  |  |  |
| Illiterate (Ref) |  |  |  |  |  |  |  |  |  |  |
| Primary (up to 5 grade) |  |  |  |  |  |  | 0.169 | 0.03, 4.41 | -0.459 | -2.60, 1.68 |
| Secondary & higher |  |  |  |  |  |  | 1.944 | -1.88, 2.22 | 1.046 | -1.29, 3.38 |
| **Marital Status** |  |  |  |  |  |  |  |  |  |  |
| Married |  |  |  |  | 4.740 | -11.38, 20.86 |  |  | 4.455 | -11.75, 20.66 |
| Unmarried (Ref) |  |  |  |  |  |  |  |  |  |  |
| **Parity** |  |  |  |  |  |  |  |  |  |  |
| Primi (Ref) |  |  |  |  |  |  |  |  |  |  |
| Multi |  |  |  |  | 1.290 | -0.80, 3.38 |  |  | 1.044 | -1.08, 3.17 |

*P<0.05
