# Supplementary material for: Do experiences and perceptions about quality of care differ among social groups in Nepal? : A study of maternal healthcare experiences of women with and without disabilities, and Dalit and non-Dalit women
Source: PLoS One. 2017 Dec 19;12(12):e0188554. doi: 10.1371/journal.pone.0188554 (PMC5736179; doi:10.1371/journal.pone.0188554)
Supplement: S1 ANNEX — (DOCX) [file pone.0188554.s006.docx]

## ANNEX 1: UN WASHINGTON GROUP DISABILITY CRITERIA (SHORT SET)

**Census Questions on Disability Endorsed by the Washington Group**

1. Do you have difficulty seeing, even if wearing glasses?

a. No - no difficulty

b. Yes – some difficulty

c. Yes – a lot of difficulty

d. Cannot do at all

2. Do you have difficulty hearing, even if using a hearing aid?

a. No- no difficulty

b. Yes – some difficulty

c. Yes – a lot of difficulty

d. Cannot do at all

3. Do you have difficulty walking or climbing steps?

a. No- no difficulty

b. Yes – some difficulty

c. Yes – a lot of difficulty

d. Cannot do at all

4. Do you have difficulty remembering or concentrating?

a. No – no difficulty

b. Yes – some difficulty

c. Yes – a lot of difficulty

d. Cannot do at all

5. Do you have difficulty (with self-care such as) washing all over or dressing?

a. No – no difficulty

b. Yes – some difficulty

c. Yes – a lot of difficulty

d. Cannot do at all

6. Using your usual (customary) language, do you have difficulty communicating, for example understanding or being understood?

a. No – no difficulty

b. Yes – some difficulty

c. Yes – a lot of difficulty

d. Cannot do at all
