# Supplementary material for: Do experiences and perceptions about quality of care differ among social groups in Nepal? : A study of maternal healthcare experiences of women with and without disabilities, and Dalit and non-Dalit women
Source: PLoS One. 2017 Dec 19;12(12):e0188554. doi: 10.1371/journal.pone.0188554 (PMC5736179; doi:10.1371/journal.pone.0188554)
Supplement: S2 ANNEX — (DOCX) [file pone.0188554.s007.docx]

## ANNEX 2: Perception Survey Tool (20-items five Rating Scale Likert Type)

| **A** | **Health Facility** |  |  |  |  |  |
| --- | --- | --- | --- | --- | --- | --- |
| 1 | **Staff Adequacy**:  In your opinion, the number of staff in HF is adequate | 1. Strongly Disagree | 1. Disagree | 1. Neutral | 1. Agree | 1. Strongly   Agree |
| 2 | **Staff availability**  In your opinion, Staff easily available for pregnancy check up and delivery at any time | 1. Strongly Disagree | 1. Disagree | 1. Neutral | 1. Agree | 1. Strongly   Agree |
| 3 | **System & Honesty: Any charges and Hidden cost**: In your opinion do HF and or its staff charges or accept some fee or any other hidden cost/kinds | 1. Strongly Disagree | 1. Disagree | 1. Neutral | 1. Agree | 1. Strongly   Agree |
| 4 | **Enough Rooms and Space:**  In your opinion, the waiting rooms, examination rooms and delivery rooms are enough in the HF | 1. Strongly Disagree | 1. Disagree | 1. Neutral | 1. Agree | 1. Strongly   Agree |
| 5 | **Equipment and Materials:**  In your opinion, the equipment and materials including beds for exam and delivery are enough and well suited for maternal pregnancy check up and delivery | 1. Strongly Disagree | 1. Disagree | 1. Neutral | 1. Agree | 1. Strongly   Agree |
| 6 | **Cleanliness & Facilities:** In your opinion, water, hand washing, toilet and beds are adequately provisioned and clean enough | 1. Strongly Disagree | 1. Disagree | 1. Neutral | 1. Agree | 1. Strongly   Agree |
| 7 | **System: HF Opening Time**  In your opinion, HF is open full time as the people’s convenience | 1. Strongly Disagree | 1. Disagree | 1. Neutral | 1. Agree | 1. Strongly   Agree |
| **B** | **Health Care Delivery** |  |  |  |  |  |
| 1 | **Service Package/standard:**  In your opinion complete services such as examination, tests, medication were given to you while receiving services | 1. Strongly Disagree | 1. Disagree | 1. Neutral | 1. Agree | 1. Strongly   Agree |
| 2 | **Capable/skilled/trained** staff: In your opinion, HF staff were not trained and skill full enough | 1. Strongly Disagree | 1. Disagree | 1. Neutral | 1. Agree | 1. Strongly   Agree |
| 3 | **Drugs/Supplies** (Enough?)  In your opinion, patients can obtain good quality drugs and supplies required timely and easily from the HF | 1. Strongly Disagree | 1. Disagree | 1. Neutral | 1. Agree | 1. Strongly   Agree |
| 4 | **Dignity/Privacy**:  Pregnancy checkups and delivery were conducted in front of others | 1. Strongly Disagree | 1. Disagree | 1. Neutral | 1. Agree | 1. Strongly   Agree |
| 5 | You feel very much un necessary and humiliating **procedure** during ante-natal and delivery care | 1. Strongly Disagree | 1. Disagree | 1. Neutral | 1. Agree | 1. Strongly   Agree |
| **C** | **Personnel/Inter-personnel Aspect** |  |  |  |  |  |
| 1 | **Open/friendly:**  In your opinion, the health staff in HF are very open and friendly | 1. Strongly Disagree | 1. Disagree | 1. Neutral | 1. Agree | 1. Strongly   Agree |
| 2 | **Compassionate** (Kind/Sympathetic)  In your opinion, the health staffs in the HF are very kind & sympathetic towards patients | 1. Strongly Disagree | 1. Disagree | 1. Neutral | 1. Agree | 1. Strongly   Agree |
| 3 | **Respectful/Welcoming**:  In your opinion, health staffs in the HF do not care and respect patients | 1. Strongly Disagree | 1. Disagree | 1. Neutral | 1. Agree | 1. Strongly   Agree |
| 4 | **Enough time for explaining / Information**  In your opinion, HF staff explain clearly giving (devoting) enough time | 1. Strongly Disagree | 1. Disagree | 1. Neutral | 1. Agree | 1. Strongly   Agree |
| **D** | **Access to Service** |  |  |  |  |  |
| 1 | **Transport Money:**  It is hard to get delivery incentive | 1. Strongly Disagree | 1. Disagree | 1. Neutral | 1. Agree | 1. Strongly   Agree |
| 2 | **Transport:**  Transportation was no problem to reach the Facility | 1. Strongly Disagree | 1. Disagree | 1. Neutral | 1. Agree | 1. Strongly   Agree |
| 3 | **Distance:**  The distance from your home to health facility is very far | 1. Strongly Disagree | 1. Disagree | 1. Agree | 1. Agree | 1. Strongly Agree |
| 4 | **Building accessible for all:**  HF building and inside arrangement is accessible to all including women with disabilities | 1. Strongly Disagree | 1. Disagree | 1. Neutral | 1. Agree | 1. Strongly   Agree |
